# Preclinical Assessment of Carboplatin Treatment Efficacy in Lung Cancer by ^18^F-ICMT-11-Positron Emission Tomography

**DOI:** 10.1371/journal.pone.0091694

**Published:** 2014-03-11

**Authors:** Timothy H. Witney, Robin R. Fortt, Eric O. Aboagye

**Affiliations:** Department of Surgery and Cancer, Imperial College London, London, United Kingdom; National Cancer Center, Japan

## Abstract

Tumour response to therapy is assessed primarily in the clinic by monitoring reductions in tumour size. However, this approach lacks sensitivity since in many cases several weeks may elapse before there is evidence of tumour shrinkage. There is therefore a need to develop non-invasive imaging techniques for monitoring tumour treatment response in the clinic. Here, we assessed the pre-clinical utility of ^18^F-ICMT-11 positron emission tomography - a method for detecting caspase 3/7 activation - in non-small cell lung cancer (NSCLC). ^18^F-ICMT-11 uptake was compared to molecular biochemical measures of cell death in PC9 and A549 NSCLC cells following treatment with carboplatin *in vitro* and *in vivo*. Carboplatin-induced apoptosis in the ERCC1 low/mutant *EGFR* PC9 cells was characterised by time and dose-related increased caspase-3/7 activation, poly-ADP-ribose polymerase cleavage and Annexin V staining. ^18^F-ICMT-11 uptake was consequently increased up to 14-fold at 200 µM carboplatin compared to vehicle treated cells (*P*<0.01). In contrast, necrosis was the predominant death mechanism in ERCC1 high/wt *EGFR* A549 cells and no change in ^18^F-ICMT-11 uptake was detected. *In vivo*, histological analysis of PC9 tumour xenografts indicated high pre-therapy necrosis. A 4.6-fold increase in cleaved caspase-3/7 was measured in non-necrotic regions of PC9 tumours at 48h post carboplatin therapy. Average PET-derived tumour ^18^F-ICMT-11 uptake was insensitive to changes in apoptosis in the presence of substantial pre-existing necrosis. PET-based voxel intensity sorting however, identified intra-tumoural regions of high ^18^F-ICMT-11 uptake, enabling accurate assessment of apoptosis and therefore therapy response. In A549 tumours that lacked high pre-therapy necrosis, carboplatin induced growth inhibition that was only minimally associated with apoptosis and thus not detectable by ^18^F-ICMT-11 PET.

## Introduction

Non-small cell lung cancer (NSCLC) accounts for the highest cancer-related mortality [1]. Adaptive randomisation of patients to different therapies on the basis of biopsy-informed tumour profiling underscores potential benefit of biomarkers in predicting response to therapy (BATTLE trial [2]). The existence of diverse resistance mechanisms in NSCLC including those driven by *EGFR*, *ELM-ALK*, and *AXL* (mutant) gene products however, implies that upfront patient stratification for therapy is problematic [3]. Therapy can lead to rapid extinction of sensitive clones but equally aggressive subclone expansion leads to transient remission and recurrence [4]. In this context several resistance mechanisms involving deregulated apoptosis pathways have been identified [3]. With a limited number of ‘life prolonging’ therapies and an incomplete understanding of drug resistance mechanisms, it is possible that early evaluation of efficacy may allow more timely switch to alternative therapies. There is however, paucity of early efficacy biomarkers. Regarding imaging efficacy biomarkers, a review by Zhao and co-workers highlighted utility of molecular imaging including positron emission tomography (PET) when combined with anatomical imaging, as a way to assess efficacy in biopsy-inaccessible lesions [5], and superior to clinical assessment by the Response Evaluation Criteria in Solid Tumours (RECIST) alone [6].

One of the well described pathway biomarkers linked to both innate and acquired resistance in NSCLC is the apoptosis pathway [3]. Apoptosis, or programmed cell death, is an essential process required for tissue homeostasis, embryonic development and the elimination of deleterious cells within the body. During tumourigenesis the mechanisms that govern normal cell apoptosis become deregulated. Some of the most commonly mutated genes found in cancer, p53 and Bcl-2, dictate if/when cells live or die [7], [8]. To overcome intrinsic tumour resistance to normal death stimuli, traditional cytotoxic, radiotherapy and mechanism-based therapies have been designed to induce tumour-specific apoptotic cell death through numerous divergent mechanisms. These divergent mechanisms converge with the activation of the effector caspase, caspase-3, during the execution phase of apoptotic cell death, with subsequent commitment to DNA degradation, breakdown of the cellular cytoskeleton, membrane blebbing, formation of apoptotic bodies and removal of the cell by the immune system [9].

Blood biomarkers of cell death have been explored in lung cancer via measurement of soluble caspase-cleaved cytokeratin 18 product M30 [10], although this methodology neither provides information on heterogeneous lesion response nor is it able to distinguish between tumour response and normal tissue toxicity. Given the almost universal occurrence of caspase-3/7 activation in programmed cell death, its detection by imaging could be a promising biomarker of treatment efficacy. We have recently developed a caspase-3/7-specific probe, ^18^F-(*S*)-1-((1-(2-fluoroethyl)-1H-[1], [2], [3]triazol-4-yl)methyl)-5-(2(2,4-difluorophenoxymethyl)-pyrrolidine-1-sulfonyl)isatin (^18^F-ICMT-11), for the *in vivo* imaging of therapy-induced tumour apoptosis. ^18^F-ICMT-11 has been shown by us and others to be a sensitive measure of both traditional cytotoxic-induced cell death [11]–[13], and tumour apoptosis following treatment with a small molecule caspase activator [11]. Automated, facile radiolabeling of ^18^F-ICMT-11 to GMP standards has been described [14], with a first-in-man study reporting favourable dosimetry profile [15]. NSCLC can present as a complex lesion including pre-therapy necrotic components; a detailed assessment of specificity of ^18^F-ICMT-11 towards apoptotic cell death in comparison to therapy-induced necrosis has not been previously reported. In this article, we present a novel strategy for the detection of treatment efficacy with ^18^F-ICMT-11 PET in preclinical models of NSCLC with varying responses to carboplatin, linked to unique genetic pre-determinants of response.

## Materials and Methods

### Cell Culture

PC9 and A549 cells were from LGC Standards (Teddington, Middlesex, UK). PC9 cells were maintained in RPMI 1640 medium, with A549s grown in DMEM. Both media were supplemented with 10% foetal calf serum, 2 mM L-glutamine, 100 U.mL−1 penicillin and 100 µg.mL−1 streptomycin (Invitrogen, Paisley, Refrewshire, UK) and cells were maintained at 37°C in a humidified atmosphere containing 5% CO_2_. Cell death was induced by the addition of carboplatin (Accord Healthcare Ltd., Middlesex, UK; 0–200 µM).

### Growth Inhibition Assay

Drug concentrations that inhibited 50% of cell growth (GC_50_) were determined using a sulphorhodamine B (SRB) technique as described elsewhere [16]. All cell lines were treated for 72 h, 24 h post seeding, unless otherwise stated.

### Flow Cytometric Measurements of Cell Death

Cells were trypsinised (0.25% trypsin; 1 mM EDTA) and harvested by centrifugation (1300 g, 3 min). Detached cells present in the media before trypsinisation were retained and pooled with the trypsinised cells. Cell pellets (1×10^6^ cells) were washed in ice-cold HEPES-buffered saline (10 mM HEPES, 140 mM NaCl, 2.5 mM CaCl_2_, pH 7.4) and resuspended in 100 µL of the same buffer. Annexin V, Alexa Fluor 488 (Invitrogen) was added (5 µL/100 µL of cell suspension) in combination with 7-Aminoactinomycin D (7-AAD; 20 µg.mL^-1^; Invitrogen), before incubating for 10 min at 20°C. The resulting mixture was washed once, kept briefly on ice, and then analysed in an LSRII Cytometer (BD Biosciences, Rockville, MD), with 20,000 cells counted per event. Residual cell debris, identified on forward (FS) and side light scattering (SS) profiles, was excluded from the analysis by selective gating.

### Western Blots

PolyADP-ribose polymerase (PARP) and caspase-3 cleavage were assessed using a standard western blotting protocol [17]. Membranes were probed using polyclonal rabbit anti-PARP1 antibody (Abcam, Cambridge, Cambridgeshire, UK; 1∶1000), a rabbit monoclonal anti-ERCC1 antibody (Cell Signaling Technology, Danvers, MA, USA; 1∶1000) and a polyclonal rabbit anti-cleaved caspase-3 antibody (Cell Signaling Technology; 1∶1000). A rabbit anti-actin antibody (Sigma-Aldrich Co. Ltd; 1∶2000) was used as a loading control. Blots were scanned (Bio-Rad GS-800 Calibrated Densitometer; Bio-Rad, Hercules, CA, USA) and signal quantification was performed by densitometry using scanning analysis software (Quantity One; Bio-Rad).

### ^18^F-ICMT-11 Radiosynthesis

^18^F-ICMT-11 synthesis and radiolabeling was performed according to previously described methodology [14]. Radiochemical purity was >98% at end of synthesis with a specific activity of 35.1±7.9 GBq/µmol (*n* = 8).

### *In vitro* ^18^F-ICMT-11 Cell Uptake

Cells (1×10^5^) were plated into 6-well plates the night prior to treatment (vehicle/carboplatin). On the day of the experiment, fresh growth medium containing 0.74 MBq ^18^F-ICMT-11 was added to individual wells. Cell uptake was measured following incubation at 37°C in a humidified atmosphere of 5% CO_2_ for 60 min. Next, cells were trypsinised (0.25% trypsin; 1 mM EDTA) and harvested by centrifugation (1300 g, 3 min). Detached cells present in the media before trypsinisation were retained and pooled with the trypsinised cells. Cells were washed 3 times with ice-cold PBS (1 mL, 1300 g, 3 min), with 20 µL subsequently taken for caspase-3/7 activity assessment (see below), prior to pelleting of the remaining cells and lysis in RIPA buffer (Thermo Fisher Scientific Inc., Rockford, IL, USA; 1 mL, 10 min). Cell lysate was transferred to counting tubes and decay-corrected radioactivity was determined on a gamma counter (Cobra II Auto-Gamma counter, Packard Biosciences Co, Pangbourne, UK). Aliquots were snap-frozen and used for protein determination following radioactive decay using a BCA 96-well plate assay (Thermo Fisher Scientific Inc., Rockford, IL, USA). Data were expressed as percent of total radioactivity per mg protein, calibrated using 10 µL standards of the 0.74 MBq/mL ^18^F-ICMT-11 stock solution.

### Caspase-3/7 Activity Assay

Caspase-3/7 activity was determined using Promega’s caspase-3/7 assay according to the manufacturer’s instructions (Promega, Madison, WI, USA). Cells were incubated for 1 h with Caspase-Glo reagent, and the enzymatic activity of caspase-3/7 was measured using a TopCount NXT microplate luminescence counter (PerkinElmer, Waltham, MA, USA) and normalised to protein content (BCA). Data was expressed as a fold-increase in caspase-3 activity over vehicle control cells.

### *In vivo* Tumour Models

All animal experiments were performed by licensed investigators in accordance with the United Kingdom Home Office Guidance on the Operation of the Animal (Scientific Procedures) Act 1986, under project licence 70/7177, and within the published guidelines for the welfare and use of animals in cancer research [18]. Tumour cells (2×10^6^ and 5×10^6^ for PC9 and A549, respectively) were injected subcutaneously on the back of female BALB/c nude mice (aged 6–8 weeks; Charles River), with animals treated with vehicle or carboplatin when the xenografts reached ∼100 mm^3^ (see below for treatment schedule). Tumour dimensions were measured periodically using a calliper and tumour volumes were calculated by the equation: volume = (π/6)×a×b×c, where a, b, and c represent three orthogonal axes of the tumour.

### PET Imaging Studies

Dynamic ^18^F-ICMT-11 imaging scans were carried out on a dedicated small animal PET scanner (Siemens Inveon PET module, Siemens Medical Solutions USA, Inc.) following a bolus *i.v.* injection of ∼3.7 MBq of the radiotracer into tumour-bearing mice [19]. Dynamic scans were acquired in list mode format over 60 min. The acquired data were then sorted into 0.5 mm sinogram bins and 19 time frames for image reconstruction (4×15 s, 4×60 s, and 11×300 s), which was done by iterative reconstruction (2D-OSEM). The Siemens Inveon Research Workplace software was used for visualization of radiotracer uptake in the tumour; 30–60 min cumulative images of the dynamic data were employed to define 3-dimensional (3D) regions of interest (ROIs). The count densities were averaged for all ROIs at each time point to obtain a time versus radioactivity curve (TAC). Tumour TACs were normalized to injected dose, measured by a VDC-304 dose calibrator (Veenstra Instruments), and expressed as percentage injected dose per mL tissue, using the calibration factor determined for the Inveon PET scanner. For image visualization, 3D-OSEM reconstruction was performed and presented as summed 30–60 min frames.

### *In vivo* Carboplatin Treatment Schedule

For treatment-response studies, mice with size-matched approximately 100 mm^3^ xenograft tumours were treated i.p. with either vehicle (saline; 0.012 mL/g body weight) or carboplatin (Accord Healthcare Ltd.; 120 mg/kg; 0.012 mL/g body weight). 24 h post injection, carboplatin-treated and vehicle control mice were imaged by ^18^F-ICMT-11 PET. A second cohort of mice received two doses of either vehicle or carboplatin, with the second injection administered 24 h after the initial dose. These mice were subsequently imaged by ^18^F-ICMT-11 PET 48 h after the initial dose.

### PET-based Voxel Intensity Sorting Histograms

The intensities of all voxels within the tumour ROIs were computed and sorted as per their intensity frequency to give the PET-based voxel intensity sorting (PVIS) histograms [11]. For each ROI, all the voxels, 30–60 minutes post radiotracer addition and their associated intensity were extracted (∼300 voxels per ROI). The voxel intensities distributions were further processed through a statistical analysis (Prism v5.0 software, GraphPad Software, San Diego, CA, USA). Within the narrow range of apoptosis seen, we arbitrary selected the 95^th^ percentile cut-off to biologically describe the 5% highest intensity voxels–likely to contain apoptotic cells – rather than, for example, on the basis of receiver operating characteristic analysis.

### Active Caspase-3 and TUNEL Immunohistochemistry Assay

Following PET imaging studies, tumour tissues were excised, fixed in formalin, embedded in paraffin, sectioned (5 µm slices) and processed for active caspase-3 and DNA degradation terminal deoxynucleotidyl transferase dUTP nick end labelling (TUNEL) fluorescent detection assays using the cleaved caspase-3 (Asp 175) monoclonal antibody (Cell Signaling Technology) coupled with the Alexa Fluor 594 goat anti-rabbit (Invitrogen) and the In Situ Cell Death Detection Kit (Roche), respectively. The ProLong Gold Antifade mounting solution (Invitrogen) containing 4′,6-diamidino-2-phenylindole (DAPI) was added to tissue sections prior to mounting of coverslips. The TUNEL assay was performed according to the manufacturer instructions, with caspase-3 staining performed according to [11], [13]. Alternate sections were counterstained with hematoxylin and eosin (H&E) staining. 10 random ‘non-necrotic’ fields per section (at 400× magnification) were captured using an Olympus BX51 fluorescent microscope for each tumour and the staining intensities (% staining per total FOV) were determined using the ImageJ software (National Institutes of Health). For PC9 sections, random FOV were selected from regions lacking extensive necrosis.

### Statistical Analysis

Data were expressed as mean ± standard deviation (SD). The significance of comparison between two data sets was determined using Student’s 2-tailed t test. ANOVA was used for multiple comparisons (Prism v5.0 software for windows, GraphPad Software). Differences between groups were considered significant if P≤0.05.

## Results

### Differential Mechanisms of Carboplatin-induced Death in PC9 and A549 Cells

**Figure 1:**
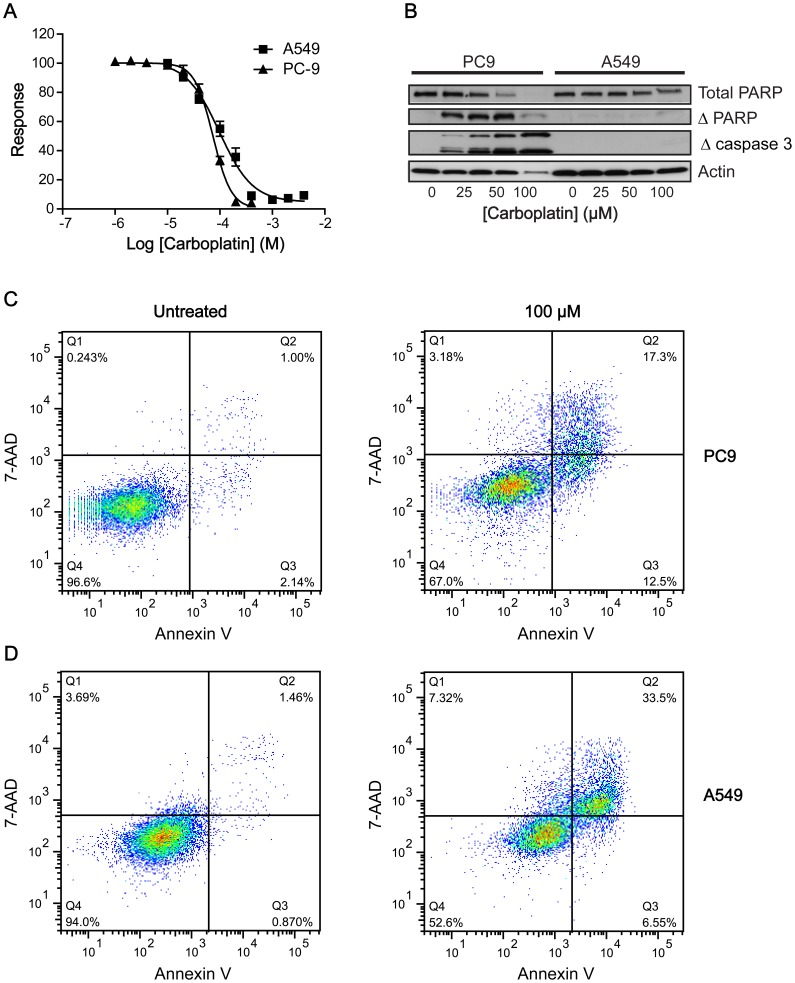
Differential responses to carboplatin treatment in PC9 and A549 cells. A: Carboplatin-induced growth inhibition in PC9 and A549 cells using a sulforhodamine B assay 72 h post treatment. B: Western blot analysis of the levels of uncleaved PARP, cleaved PARP and cleaved (active) caspase 3 72 h post carboplatin treatment (0–200 µM) in PC9 and A549 cells. Actin was used as a loading control. C, D: Flow cytometric analysis of PC9 (C) and A549 cells (D) treated with carboplatin (100 µM) or vehicle. Apoptotic cells were identified by Annexin V-Alexafluor488 (λ Ex/Em = 495/519 nm) and necrotic cells by 7-AAD (λ Ex/Em = 546/647 nm). Population Q4 represents viable cells, whereas population Q3 represents apoptotic cells that have low 7-AAD fluorescence and stain with Annexin V. Population Q2 represents secondary apoptotic/necrotic cells.

PC9 and A549 human NSCLC cells were selected for their unique genetic pre-determinants of response: The former is characterized by low DNA-damage repair protein ERCC1 expression and a mutation in *EGFR* (15 bp del of exon 19), with both characteristics independently capable of sensitizing cells to platinum-based therapies [20], [21]. In contrast, A549 cells have high ERCC1 expression (low- and high-expressing for PC9 and A549 respectively; Online Resource 1) and have wt *EGFR*. Cell death was induced *in vitro* in PC9 and A549 human NSCLC cells following carboplatin treatment (0–200 µM). Dose-dependent growth inhibition evaluated at 72 h post treatment by a sulforhodamine B assay (SRB) showed half maximal growth inhibition (GC_50_) of 71.6±9.5 µM and 136±31.6 µM for PC9 and A549 respectively (*n* = 3; Fig. 1A). Apoptotic cell death was evaluated by western blotting. Levels of cleaved caspase-3 and the cleavage of its down-stream substrate, PARP, showed a dose-related increase in PC9 cells, whereas no changes were observed with A549 (Fig. 1B). Flow cytometric measurements confirmed an apoptotic mechanism of cell death in PC9s (Fig. 1C), with necrosis the primary mechanism of death in A549s (Fig. 1D).

### ^18^F-ICMT-11 Cell Uptake Correlates with Caspase-3 Activation *in vitro*

#### Dose-dependent changes

**Figure 2:**
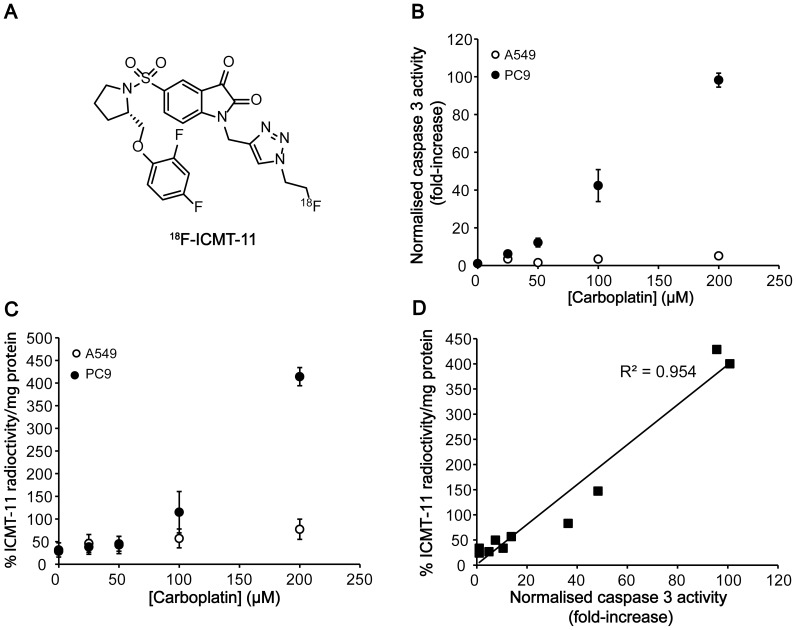
^18^F-ICMT-11 cell uptake correlates to dose-dependent increases in caspase 3 activity following carboplatin treatment. A: Chemical structure of ^18^F-ICMT-11. B: Dose-dependent changes in caspase 3/7 activity following carboplatin treatment. C: Dose-dependent changes in ^18^F-ICMT-11 uptake in cells following carboplatin treatment. D: Correlation between caspase 3 activity and ^18^F-ICMT-11 uptake in PC9 cells.

Carboplatin-induced cell death was initially evaluated with ^18^F-ICMT-11 *in vitro* (Fig. 2A). Carboplatin treatment of PC9 cells resulted in a dose-dependent activation of caspase-3/7 activity (Caspase-Glo assay; Fig. 2B), up to 87±19-fold at 200 µM (*P* = 0.001, *n* = 3). These data further supports results obtained by western blot and flow cytometry (Fig. 1B and C respectively). Changes in caspase-3/7 activity were not detected in A549 at similar drug concentrations (Fig. 2B).

Addition of 0.74 MBq (20 µCi) ^18^F-ICMT-11 to cells 72h post carboplatin treatment (0–200 µM) resulted in detectable uptake and retention of the radiotracer following 1 h pulse-chase with the radiotracer. An increase in cellular uptake, proportional to carboplatin dose was measured in PC9 cells; reaching statistical significance at 100 µM, increasing from 28.8%±6.7% radioactivity/mg protein for vehicle-treated controls to 414.4%±20.1% radioactivity/mg protein at 200 µM (*n* = 3; P<0.01), a 14.4-fold increase (Fig. 2C). There was an excellent correlation between cellular caspase-3/7 activity and ^18^F-ICMT-11 uptake in this line (Fig. 2D; R^2^ = 0.954). No significant change in ^18^F-ICMT-11 uptake was detected with A549 cells following addition of carboplatin (Fig. 2C).

#### Time course of apoptotic cell death

**Figure 3:**
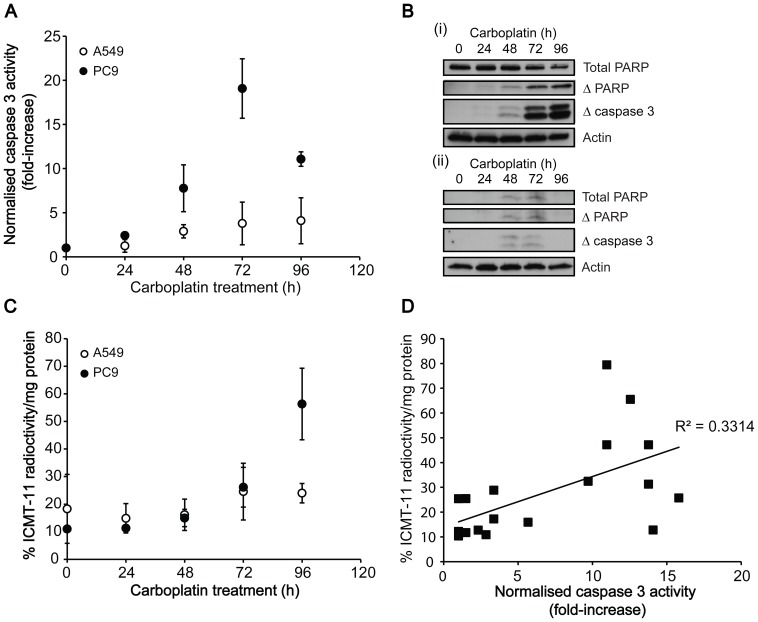
Temporal changes in cell death markers and ^18^F-ICMT-11 uptake after carboplatin treatment. A: Time course of changes in caspase 3/7 activity following carboplatin treatment. B: Western blot analysis of the levels of uncleaved PARP, cleaved PARP and cleaved (active) caspase 3 post 50 µM carboplatin treatment (0–96 h) in PC9 (i) and A549 cells (ii). C: Temporal changes in ^18^F-ICMT-11 uptake in cells following carboplatin treatment. D: Correlation between caspase 3 activity and ^18^F-ICMT-11 uptake in PC9 cells.

The time course of apoptotic cell death was further evaluated at 50 µM carboplatin, a dose close to the GC_50_ of PC9 cells (Fig. 1A). The onset of apoptosis in PC9s was detectable at 48 h after treatment, measured by a 7.8±4.6-fold increase in caspase-3/7 activity (*P* = 0.03; *n* = 4; Fig. 3A), with caspase-3 and PARP cleavage also evident by western blot (Fig. 3B(ii)). A temporal increase in cleaved caspase-3 was detected up to 96 h post treatment in PC9 cells however, there was a reduction in caspase-3 activity between 72 h and 96 h, falling from 19.1±3.4-fold to 11.1±0.8-fold increase over baseline, respectively (*n* = 4; *P* = 0.036). The magnitude of apoptotic response was 7.9-fold lower with cells treated with 50 µM for 96 h in comparison to a concentration of 200 µM at the same time point. ^18^F-ICMT-11 intracellular accumulation mirrored the temporal increase of cleaved caspase-3 in this cell line (Fig. 3B, C), rising from 15.8%±4.2% radioactivity/mg protein to 64%±8.1% radioactivity/mg protein in cells treated at 50 µM for 96 h in comparison to untreated control cells (*P* = 0.009). Despite excellent correlation between cellular cleaved caspase-3 and ^18^F-ICMT-11 uptake in this cell line, correlation between ^18^F-ICMT-11 uptake and caspase-3/7 activity was less well defined (Fig. 3D; R_2_ = 0.3314). Despite the detection of faint bands corresponding to cleaved caspase-3 and PARP with A549 cells treated either 48 h or 72 h (Fig. 3B (ii)), there was no increase in detectable caspase-3/7 activity (Fig. 3A). In parallel, there was no significant change in ^18^F-ICMT-11 over the entirety of this time course (Fig. 3C).

### ^18^F-ICMT-11 can Distinguish Apoptotic from Necrotic Cell Death *in vivo*

#### Temporal changes in treatment response

**Figure 4:**
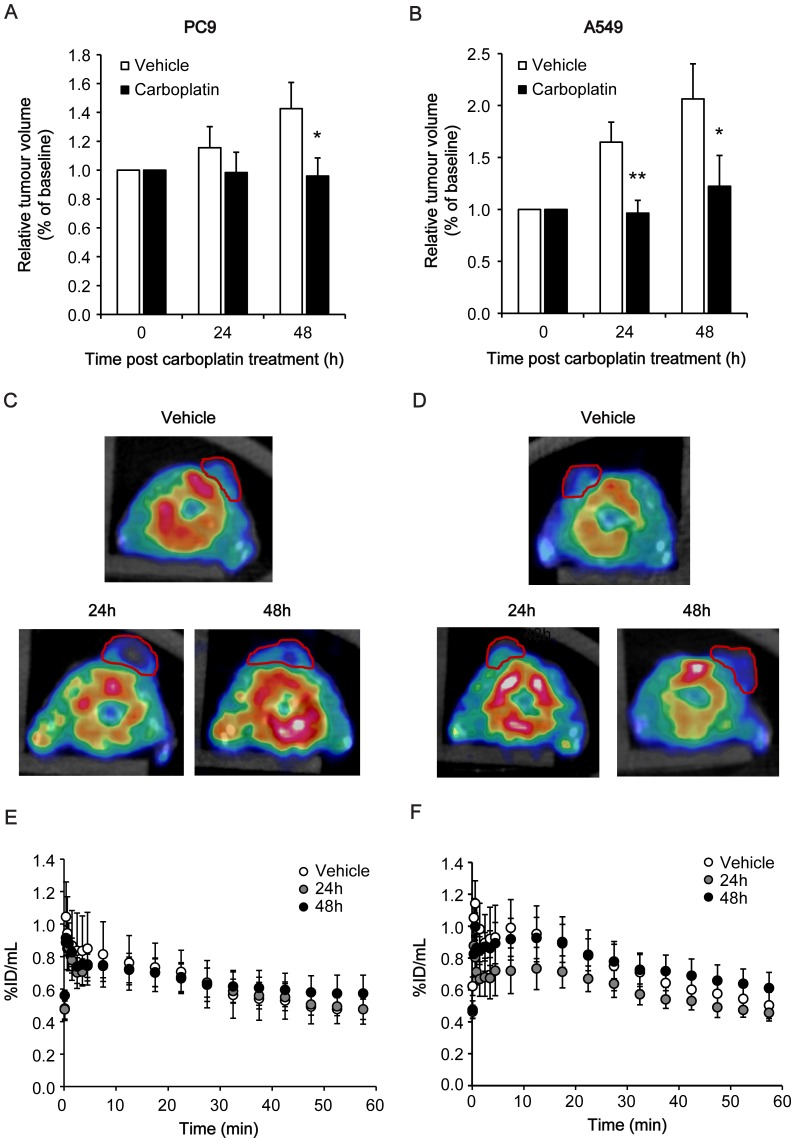
^18^F-ICMT-11 PET image analysis of PC9 and A549 xenografts in vehicle and carboplatin-treated mice. A, B: Tumour volumes recorded by calliper measurements of PC9 (A) and A549 tumours (B) pre- and post-carboplatin treatment as indicated. Data shown are mean ± SD of % volume compared to baseline (*n* = 4). *, *P*<0.05; **, *P*<0.01. C, D: Representative axial PET-CT images (30–60 min summed activity) for PC9 (C) and A549 (D) tumours. Tumour margins, indicated from CT image, are outlined in red. Mean ± SD (*n* = 4–6 animals per group). E, F: The tumour TAC representing average counts from a dynamic 60-minute scan for PC9 (E) and A549 xenografts (F) following carboplatin treatment (vehicle, 24 h or 48 h carboplatin-treated; *n = *4–6 animals per group).

PC9 and A549 tumours were grown as xenografts, with calliper measurements of tumour size measured after vehicle, 24 h and 48 h carboplatin treatment (120 mg/kg i.p. daily) and compared to baseline. For both tumours, carboplatin treatment resulted in growth arrest. For PC9 tumours, a significant difference in tumour volume was measured 48 h post carboplatin treatment in comparison to vehicle controls, which increased to 143±18% baseline volume, with carboplatin-treated tumours remaining at 96±13% baseline volume (*P* = 0.013; *n* = 4; Fig. 4A). A significant delay in tumour growth was measured both 24 h and 48 h post carboplatin treatment in A549 tumours in comparison to vehicle controls (Fig. 4B); later time points were not measured.

We next assessed ^18^F-ICMT-11 as a sensitive marker of tumour cell death *in vivo*. Tumour-associated ^18^F-ICMT-11 radioactivity was determined by dynamic 60-minute PET imaging. Representative axial images depicting tumour-associated ^18^F-ICMT-11 are illustrated in Figures 4C and 4D for PC9 and A549 tumours, respectively. 3D regions of interest were defined for both PC9 and A549 tumours, with average counts used to obtain a time versus radioactivity curve (ROI; Fig. 4E and 4F respectively). For both tumour lines, there was no significant difference in averaged tumour-associated ^18^F-ICMT-11 radioactivity at 24 h and 48 h post carboplatin treatment in comparison to vehicle controls as defined by the area under the TAC (30–60 min) or normalized uptake values at 60 min post radiotracer injection (%ID/mL_60_).

#### Confounds of tumour heterogeneity

**Figure 5:**
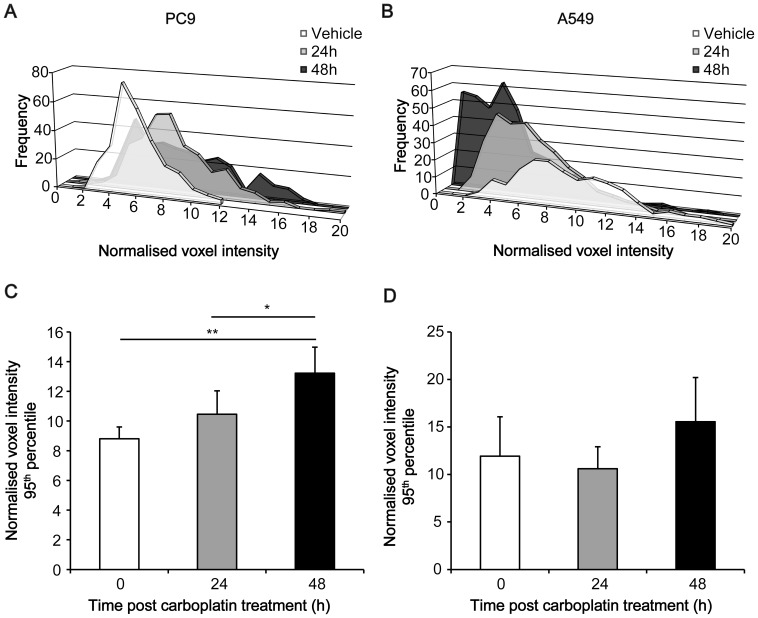
Voxel-wise analysis of ^18^F-ICMT-11 PET imaging data by PVIS. The intensities of all voxels within the tumour ROIs were computed and expressed as histogram plots of normalized voxel intensity versus the number of voxels. A, B: Typical data from three representative animals (vehicle, 24 h or 48 h carboplatin-treated) for PC9 (A) and A549 (B) are shown. C, D: The statistical comparison of 95^th^ percentile voxel intensities for PC9 (C) and A549 (D) was performed using Prism v5.0 software (GraphPad). Mean ± SD (*n* = 4–6 animals per group).*, *P*<0.05; **, *P*<0.01.

Axial images of 30–60 min summed activity revealed a heterogeneous pattern of ^18^F-ICMT-11 distribution in PC9 tumours (Fig. 4C), whereas A549 radioactivity was more homogeneous in its distribution (Fig. 4D). Although the partial volume effect may contribute to this apparent heterogeneity, voxel-wise analysis of the PET data by PVIS (30–60 min) confirmed non-uniform distribution of ^18^F-ICMT-11 tumour radioactivity (Fig. 5A and 5B for PC9 and A549 respectively). For PC9s, there was a clear shift in PVIS histograms over the 48 h time course, with a 1.5-fold group average increase in the number of voxels with high intensity in PC9 tumour ROIs of carboplatin injected mice compared to vehicle, as depicted by the 95^th^ percentile (*P* = 0.01; Fig. 5C). No significant difference voxel intensities or distribution were observed in A549 tumours over the treatment time course (Fig. 5D).

**Figure 6:**
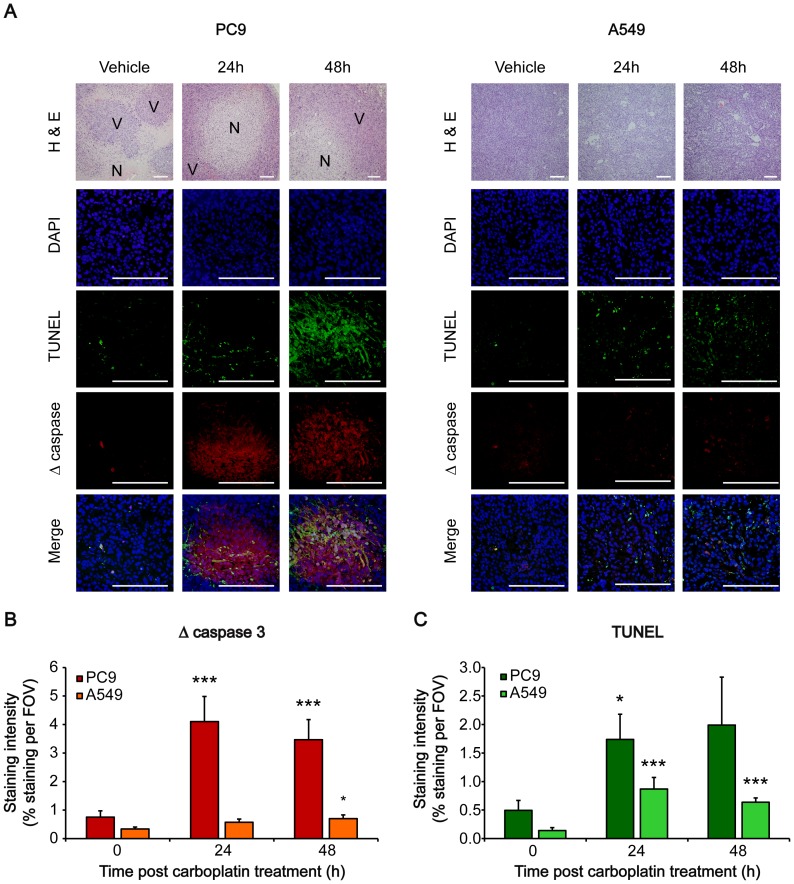
Tumour active caspase-3 and TUNEL immunohistochemistry analysis. Tumour tissues were removed after PET imaging scan, processed for histological analysis and stained for active (cleaved) caspase-3 and DNA fragmentation (TUNEL assay) detection, in conjunction with H&E staining. A: Representative images of histological tumour sections are shown. Staining intensities for cleaved caspase 3 (B) and TUNEL (C) were determined using the ImageJ software and expressed as percent staining per field. Data are mean ± SD. *, *P*<0.05; ***, *P*<0.001. *n* = 3 tumour sections with 5 random FOV per section. Photographic images of H&E-stained sections were acquired at 100×, with all other images acquired at 400×. Scale bar = 200 µm. Abbreviations: N, necrotic; V, viable.

H&E staining of formalin-fixed tumours confirmed PC9 tumour heterogeneity, characterized by extensive regions of pre-existing necrosis prior to treatment (Fig. 6A) consistent with that of human lung cancer patient samples [22]. In the non-necrotic, ‘healthy’ regions of PC9 tumours, carboplatin treatment significantly increased apoptosis, defined by an increase in cleaved caspase-3 and TUNEL staining (Fig. 6B and 6C respectively). Cleaved caspase-3 staining of PC9 tumour sections was 5.4-fold higher following carboplatin treatment, from 0.76%±0.22% in vehicle controls, to 4.11%±0.88% staining 24 h post carboplatin treatment (*P* = 0.002); 3.5%±0.70% cleaved caspase-3 staining was measured 48 h post treatment, significantly higher than vehicle controls (4.6-fold increase; *P* = 0.0003; Fig. 6B). In comparison to vehicle-treated control PC9 tumours, TUNEL staining was 3.5-fold higher 24 h post treatment, rising from 0.49%±0.17% staining to 1.74%±0.17%, (*P* = 0.047; Fig. 6C). No significant change in TUNEL staining was measured 48 h post treatment due to large variations in the randomly selected FOVs. In A549 tumours, carboplatin treatment resulted in loss of cellularity, defined by H&E staining, and higher TUNEL-positive cells; increasing from 0.14%±0.05% to 0.87%±0.02% in vehicle and 24 h carboplatin-treated tumours, respectively (*P* = 0.0015; Fig. 6A & 6C). A small elevation in cleaved caspase-3 staining was measured 48 h post carboplatin treatment in A549 tumours, increasing from 0.34%±0.07% in vehicle-treated tumours to 0.70±0.13% (2-fold increase; *P* = 0.02; Fig. 6B).

## Discussion

Platinum-based therapy remains the most effective therapeutic regimen for advanced NSCLC with response rates of 15–30% in unselected patients (median survival, 10–12 months) [23]. Several mechanisms have been described to underscore innate and acquired resistance to platinum-based therapy involving alterations in nucleotide excision repair [21]. ERCC1 expression plays a central role in these DNA damaged-mediated repair pathways [24]. More recently, other mechanisms involving *EGFR* have been described. In particular, epistatic interactions between FANCD2 and mutant *EGFR* cells have been shown to impair homologous recombination repair and sensitize cells to platinum-based therapy [20]. Currently, evaluation of efficacy to platinum-based therapy, as well as most other therapies rely on RECIST evaluation, although many months may pass before treatment failure is detected by RECIST criteria alone [25]. A sensitive method to longitudinally monitor patient response to current therapies is therefore required to accurately assess treatment efficacy for this disease.

Mechanistically, down-regulation of the apoptotic response underlies resistance of lung cancer to platinum and other evolving new therapies for lung cancer [3] and thus apoptosis biomarkers could be useful for assessing efficacy. Regarding imaging of apoptosis, a number of novel imaging strategies have been developed and two of these, ^18^F-ML-10 and ^99m^Tc-Annexin V, have progressed to clinical trials [26], [27]. ^18^F-ML-10 uptake correlates with membrane depolarization and intracellular acidification, indicative of the apoptotic response [28] however, questions relating to specificity of trapping in apoptotic cells alone with ^18^F-ML-10 still remain. Extensive studies have demonstrated utility for Annexin V to report cell death, but poor biodistribution of ^99m^Tc-Annexin V has prevented further clinical development of this radiotracer. The potential to longitudinally monitor cytotoxic-induced cell death with ^18^F-labeled Annexin V has been shown, although the small magnitude of changes in tracer uptake post-therapy demonstrated despite large reductions in tumour size [29], and the high non-specific binding of Annexin V to viable tumour cells [30] may further limit its clinical utility. Numerous studies have demonstrated that reduced ^18^F-2-Fluoro-2-deoxy-D-glucose (^18^F-FDG) uptake can identify early treatment response in tumours, including in NSCLC [31]. Changes in FDG uptake post therapy are indirectly linked to *bona fide* cell death and are known to result from alterations in the plasma membrane expression of the glucose transporters, which govern cell uptake, in combination with the loss of cellularity [17]. High uptake by infiltrating immune cells can also mask the decreased uptake by the dying tumour cells [32]. There is therefore a need to develop new imaging methods to detect treatment efficacy.

We have previously described ^18^F-ICMT-11 as a sensitive marker of chemotherapy-induced cell death in preclinical models of lymphoma [12], [13], breast and colon cancer, consistent with measured reductions in ^18^F-FDG uptake [11]. ^18^F-ICMT-11 binds to the apoptotic effector caspase, caspase-3, with sub nM affinity [33]; however, we have yet to show specificity of ^18^F-ICMT-11 to measure apoptotic cell death over necrotic mechanisms. Here we show that an increase in ^18^F-ICMT-11 uptake excellently correlates with a dose-dependent induction of caspase-3/7 activity, poly-ADP-ribose cleavage and consequent activation of apoptotic cell death in the PC9 cell line. In A549 cells, cell death was induced via the necrotic pathway, concurrent with minimal ^18^F-ICMT-11 uptake, indicating great specificity of ^18^F-ICMT-11 to trace apoptotic, but not necrotic mechanisms of cell death. Such ‘true negative’ preclinical findings are essential to further understand ^18^F-ICMT-11’s mechanism of action prior to progression to advanced clinical trials.

Time course evaluation of carboplatin treatment in PC9 cells (50 µM: ∼GC_50_) revealed a temporal increase in caspase-3 activation up to 72 h, with a reduction observed at 96 h. An increase in ^18^F-ICMT-11 paralleled the temporal increase in caspase-3 activity up to 72 h; however, a further increase in ^18^F-ICMT-11 was measured 96 h post treatment. At this time point there was a disconnect between ^18^F-ICMT-11 cellular retention, caspase-3 cleavage, and caspase-3 activity, with ^18^F-ICMT-11 uptake mirroring caspase-3 cleavage of but not residual caspase-3 activity in this late apoptotic/secondary necrotic phase (Fig. 3). We speculate that inactivated, yet cleaved caspase 3 remains in the late apoptotic cells, shown here by western blotting, permitting ICMT-11 binding and detection by PET. Ultimately, these findings provide hope that elevated ^18^F-ICMT-11 tumour uptake may persist after the transitory window of therapy-induced caspase-3 activation.

We hypothesize that differences in the intrinsic DNA-repair pathways and *EGFR* mutation status in these cells controls cell fate following carboplatin treatment [21]. Elevated ERCC1 expression in tumours, shown here in A549s in comparison to PC9s (Supporting Figure S1), is known to mediate resistance to platinum therapy [34] and may account for the absence of an apoptotic response measured here in A549s both *in vitro* and *in vivo*. Under this scenario, it is easy to envisage hyper-activation of other members of the DNA repair program, such as PARP. PARP hyperactivation in the absence of caspase activity, known to result in PARP inactivation [35], leads to rapid depletion of the NAD(H) coenzyme pool, consequent depletion of intracellular ATP and ultimately cellular necrosis – a programmed series of events, termed necroptosis [36]. As demonstrated here *in vitro*, and to some extent *in vivo*, delayed carboplatin-induced necroptosis in A549s is the predominant mechanism of cell death. Under these conditions, pure apoptosis imaging biomarkers, such as ^18^F-IMCT-11, will miss these responses to therapy. Direct and indirect readouts of therapy-induced necrosis by hyperpolarized ^13^C_2_-fumarate [37], [38] and diffusion-weighted MRI [39], [40], respectively, may therefore provide complementary readouts to response-monitoring with^18^F-ICMT-11.

*In vivo* analysis of ^18^F-ICMT-11 uptake in both PC9 and A549 xenograft-bearing mice here reflected the pattern of response observed in cells. In both tumour xenografts, *i.p.* carboplatin treatment induced growth cytostasis, measured up to 48 h post initial treatment, when compared to vehicle treatment alone (Fig. 4A & 4B). A549 tumour growth arrest was associated with a loss of tumour cellularity, defined by H&E staining of histological sections, increased DNA fragmentation, measured by TUNEL, yet minimal changes in cleaved caspase-3 staining. Although DNA fragmentation is typically thought to occur downstream of apoptosis, necroptosis is also known to elicit caspase-independent, large-scale DNA fragmentation [41]. No change in tumour-associated ^18^F-ICMT-11 activity was detected in A549 tumours following carboplatin therapy, reflecting the negligible apoptotic response to this drug in this model.

In PC9 xenografts, large pre-existing necrotic regions in the tumour prevented the differentiation between carboplatin-treated and untreated animals by average tumour-associated counts alone (Fig. 4E). The small percentage of viable tumour cells observed here may explain the relatively slow growth of these tumours when compared to A549 xenografts (Fig. 4A & B). In regions of the tumour where substantial necrosis was not observed, carboplatin treatment resulted in elevated cleaved caspase-3 and DNA fragmentation as detected by immunofluorescence, typical of an apoptotic mechanism of cell death. To capture and measure these heterogeneous regions of therapy-induced cell death with ^18^F-ICMT-11, we employed voxel intensity sorting and statistical analysis (PVIS) to identify activated caspase-3 foci. Histogram analysis of increased ^18^F-ICMT-11 voxel intensities paralleled carboplatin-induced tumour apoptosis in heterogeneous PC9 xenografts *in vivo*, whereas there was no change in ^18^F-ICMT-11 voxel distribution in treated A549 tumours vs. controls measured with this technique.

In conclusion, we demonstrate that apoptotic, but not necrotic responses of NSCLC to platinum-based therapy are detectable by ^18^F-ICMT-11. These results further establish ^18^F-ICMT-11 as a good pharmacodynamic marker of apoptosis and biomarker of efficacy, shown here even in the absence of tumour shrinkage. For analysis of heterogeneous tumours with existing necrotic regions, histogram analysis of voxel intensities enables differentiation between treated and untreated tumours, not detectable by average tumour uptake values alone. Alternative imaging strategies are required for treatment response monitoring where the primary mechanism of tumour cell death is necrosis.

## Supporting Information

Figure S1 **Western blot analysis of the levels of the DNA-damage repair protein ERCC1 in PC9 and A549 cells.** Actin was used as a loading control.
(TIF)
