# Correction: Preclinical Assessment of Carboplatin Treatment Efficacy in Lung Cancer by ^18^F-ICMT-11-Positron Emission Tomography

**DOI:** 10.1371/journal.pone.0100020

**Published:** 2014-06-06

**Authors:** The PLOS ONE Staff

The second author’s name is incorrect. The correct name is Robin Fortt. The correct citation is: Witney TH, Fortt R, Aboagye EO (2014) Preclinical Assessment of Carboplatin Treatment Efficacy in Lung Cancer by ^18^F-ICMT-11-Positron Emission Tomography. PLoS ONE 9(3): e91694. doi:10.1371/journal.pone.0091694

Figure 1 is also incorrect. In Figure 1B, the concentrations of drug do not correspond to the associated protein band. The authors have provided a corrected version of Figure 1, which can be viewed here.

**Figure 1:**
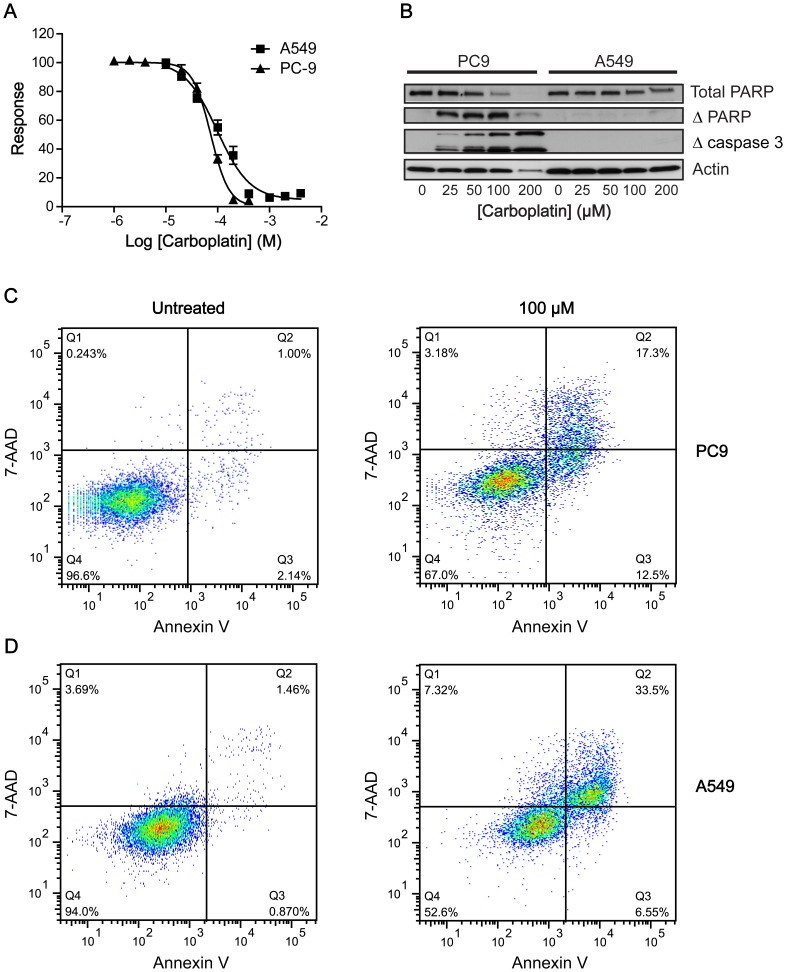
Differential responses to carboplatin treatment in PC9 and A549 cells. A: Carboplatin-induced growth inhibition in PC9 and A549 cells using a sulforhodamine B assay 72 h post treatment. B: Western blot analysis of the levels of uncleaved PARP, cleaved PARP and cleaved (active) caspase 3 72 h post carboplatin treatment (0–200 µM) in PC9 and A549 cells. Actin was used as a loading control. C, D: Flow cytometric analysis of PC9 (C) and A549 cells (D) treated with carboplatin (100 µM) or vehicle. Apoptotic cells were identified by Annexin V-Alexafluor488 (λ Ex/Em  =  495/519 nm) and necrotic cells by 7-AAD (λ Ex/Em  =  546/647 nm). Population Q4 represents viable cells, whereas population Q3 represents apoptotic cells that have low 7-AAD fluorescence and stain with Annexin V. Population Q2 represents secondary apoptotic/necrotic cells.
