# Correction: Preclinical Assessment of Carboplatin Treatment Efficacy in Lung Cancer by ^18^F-ICMT-11-Positron Emission Tomography

**DOI:** 10.1371/journal.pone.0235804

**Published:** 2020-07-09

**Authors:** Timothy H. Witney, Robin Fortt, Eric O. Aboagye

Following the publication and correction of this article [1, 2], an error in Fig 3B(ii) has been identified whereby the wrong panel for ‘Total PARP’ was used inadvertently. The updated figure presents the correct ‘Total PARP’ panel for Fig 3B(ii), and the original, uncropped blots supporting the results in Fig 3B(ii) as well as the individual level data supporting Fig 3A, 3C and 3D have been uploaded as Supporting Information files.

**Fig 3:**
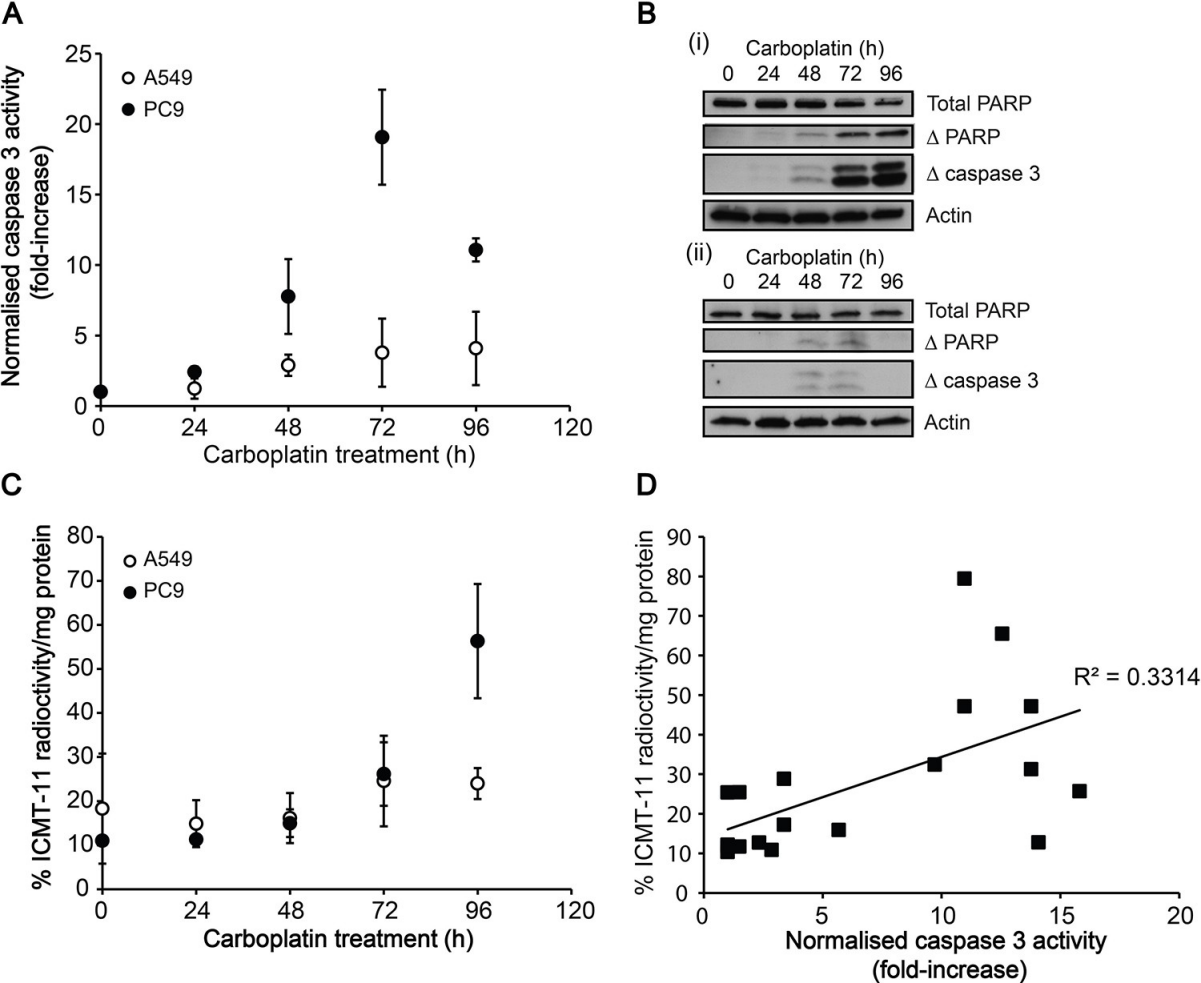
Temporal changes in cell death markers and ^18^F-ICMT-11 uptake after carboplatin treatment. A: Time course of changes in caspase 3/7 activity following carboplatin treatment. B: Western blot analysis of the levels of uncleaved PARP, cleaved PARP and cleaved (active) caspase 3 post 50 μM carboplatin treatment (0–96 h) in PC9 (i) and A549 cells (ii). C: Temporal changes in ^18^F-ICMT-11 uptake in cells following carboplatin treatment. D: Correlation between caspase 3 activity and ^18^F-ICMT-11 uptake in PC9 cells.

## Supporting information

S1 Fig Original Actin, Cleaved Caspase 3, and PARP blots underlying Fig 3B(ii). (TIF)

S1 Table Individual level data underlying Fig 3A: Time course of changes in caspase 3/7 activity following carboplatin treatment. (XLSX)

S2 Table Individual level data underlying Fig 3C: Temporal changes in ^18^F-ICMT-11 uptake in cells following carboplatin treatment. (XLSX)

S3 Table Individual level data underlying Fig 3D: Correlation between caspase 3 activity and ^18^F-ICMT-11 uptake in PC9 cells. (XLSX)
