# Supplementary figures and images for: Preclinical Assessment of Carboplatin Treatment Efficacy in Lung Cancer by 18F-ICMT-11-Positron Emission Tomography
Source: PLoS One. 2014 Mar 11;9(3):e91694. doi: 10.1371/journal.pone.0091694 (PMC3950258; doi:10.1371/journal.pone.0091694)

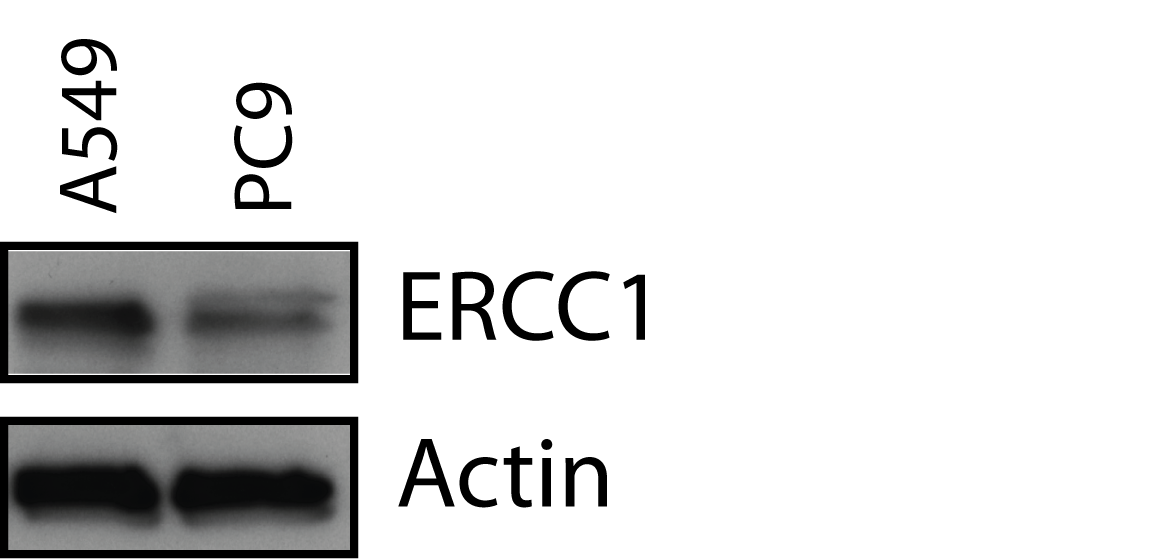

Supplement: Figure S1 — Western blot analysis of the levels of the DNA-damage repair protein ERCC1 in PC9 and A549 cells. Actin was used as a loading control. (TIF) [file pone.0091694.s001.tif]
