# Supplementary figures and images for: Correction: Preclinical Assessment of Carboplatin Treatment Efficacy in Lung Cancer by 18F-ICMT-11-Positron Emission Tomography
Source: PLoS One. 2020 Jul 9;15(7):e0235804. doi: 10.1371/journal.pone.0235804 (PMC7347147; doi:10.1371/journal.pone.0235804)

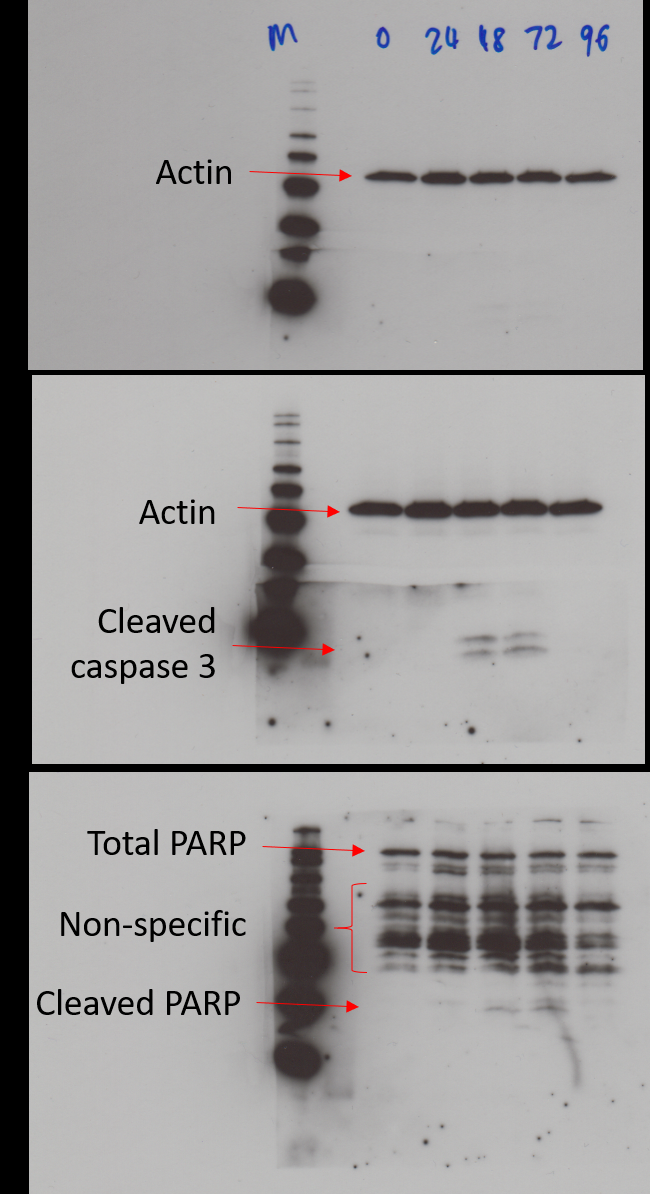

Supplement: S1 Fig — (TIF) [file pone.0235804.s001.tif]
